# The Development of a Web-Based, Patient-Centered Intervention for Patients With Chronic Myeloid Leukemia (CMyLife): Design Thinking Development Approach

**DOI:** 10.2196/15895

**Published:** 2020-05-15

**Authors:** Geneviève ICG Ector, Peter E Westerweel, Rosella PMG Hermens, Karin AE Braspenning, Barend CM Heeren, Oscar MF Vinck, Jan JM de Jong, Jeroen JWM Janssen, Nicole MA Blijlevens

**Affiliations:** 1 Department of Hematology Radboud University Medical Center Nijmegen Netherlands; 2 Department of Hematology Albert Schweitzer Hospital Dordrecht Netherlands; 3 Department of IQ Healthcare Radboud University Medical Center Nijmegen Netherlands; 4 REshape Innovation Center Radboud University Medical Center Nijmegen Netherlands; 5 Dutch Patient Advocacy Group 'Hematon' Utrecht Netherlands; 6 7 Department of Hematology Cancer Center Amsterdam Vrije Universiteit Amsterdam, Amsterdam University Medical Centers Amsterdam Netherlands

**Keywords:** eHealth, chronic myeloid leukemia, patient participation, mobile apps

## Abstract

**Background:**

With the global rise in chronic health conditions, health care is transforming, and patient empowerment is being emphasized to improve treatment outcomes and reduce health care costs. Patient-centered innovations are needed. We focused on patients with chronic myeloid leukemia (CML), a chronic disease with a generally good long-term prognosis because of the advent of tyrosine kinase inhibitors. However, both medication adherence by patients and guideline adherence by physicians are suboptimal, unnecessarily jeopardizing treatment outcomes.

**Objective:**

The aim of this study was to develop a patient-centered innovation for patients with CML using a design thinking methodology.

**Methods:**

The 5 phases of design thinking (ie, empathize, define, ideate, prototype, and test) were completed, and each phase started with the patient. Stakeholders and end users were identified and interviewed, and observations in the care system were made. Using tools in human-centered design, problems were defined and various prototypes of solutions were generated. These were evaluated by patients and stakeholders and then further refined.

**Results:**

The patients desired (1) insights into their own disease; (2) insights into the symptoms experienced, both in terms of knowledge and comprehension; and (3) improvements in the organization of care delivery. A web-based platform, CMyLife, was developed and pilot-tested. It has multiple features, all targeting parts of the bigger solution, including a website with reliable information and a forum, a guideline app, personal medical records with logs of symptoms and laboratory results (including a molecular marker and linked to the guideline app), tailored feedback based on the patients’ symptoms and/or results, screen-to-screen consulting, delivery of medication, and the collection of blood samples at home.

**Conclusions:**

The multifeatured innovation, CMyLife, was developed in a multidisciplinary way and with active patient participation. The aim of developing CMyLife was to give patients the tools to monitor their results, interpret these results, and act on them. With this tool, they are provided with the know-how to consider their results in relation to their personal care process. Whether CMyLife achieves its goal and the evaluation of the added value will be the focus of future studies. CML could become the first malignancy for which patients are able to monitor and manage their disease by themselves.

## Introduction

### Background

In 2002, the World Health Organization reported about expecting dramatic changes in global health because of the rising prevalence of chronic health conditions, underscoring the necessity for innovations in health care to effectively manage long-term health problems [1], which also requires a shift in the traditional roles of physicians and patients. The growing involvement of patients in their own care process and self-management needs to be stimulated [2] as this could improve the health outcomes of treatment and reduce health care costs [3]. Ideally, care for chronic illnesses should be patient-centered, resulting in an increased understanding and awareness of health, treatment options, symptoms, and behaviors [4,5]. This patient-centeredness should be of major focus when facilitating innovation in health care. In other sectors, involving the end user of a product in its development is a common practice, and different kinds of human-centered approaches are available [6]. One of these approaches is *design thinking*. Although a universal definition is missing, the solution-focused methodology is best described as “a discipline that uses the designer’s sensibility and methods to match people’s needs with what is technologically feasible and what a viable business strategy can convert into customer value and market opportunity” [7]. By engaging the end user from the beginning of the process, solutions are developed bottom-up instead of top-down [8]. Nowadays, design thinking is also gaining ground in the field of health care and health care education [9-13]. Design thinking and user-centered design are quite similar and start from the same standpoint—the user. The steps of both processes are quite comparable. However, design thinking emphasizes the ideation phase and focuses on finding solutions and has a specific set of tools.

### Objective

This paper aimed to describe the development and pilot testing of a patient-centered innovation that can change the current model of health care. As starting with small and rapid prototyping is key, we started with changing care for a specific group of patients (ie, patients with chronic myeloid leukemia [CML]). This malignant disease results from a reciprocal translocation between chromosomes 9 and 22, generating an abnormal Philadelphia chromosome. The resulting *BCR-ABL1* fusion gene encodes for a constitutively active tyrosine kinase, which is pivotal in the pathogenesis of CML and can be inhibited by tyrosine kinase inhibitors (TKIs). With the advent of TKIs, CML became a chronic disease rather than an often fatal one [14,15]. As with many chronic diseases, therapy compliance and guideline adherence are challenging. The paramount step in the monitoring of patients with CML is the quantification of *BCR-ABL1* transcripts with a polymerase chain reaction on peripheral blood, which represents disease activity [16]. The recommendations for the monitoring of patients with CML are defined in international guidelines [16,17]. However, guideline adherence by physicians is often suboptimal [18-20], which is associated with a higher risk of progression to an advanced phase of the disease and death [21]. Another challenge in CML care comprises suboptimal adherence to TKIs by patients, which in part relates to the adverse events (AEs) caused by TKIs [18,21-24]. Common AEs are muscle aches and cramps, fatigue, gastrointestinal symptoms, and fluid retention, among others [25,26]. AEs are the main cause of intentional nonadherence. The adequate management of AEs is a necessity, as an adherence rate of ≤90% is clearly related to a lower probability of reaching important treatment goals [22]. Furthermore, physicians tend to underestimate the severity of AEs experienced by patients and overestimate the patients’ overall health status [27].

By transforming CML care into a patient-centered care model, the self-management of monitoring and intake of TKI can be stimulated, resulting in improved health outcomes and the involvement of patients in their own care process. Hence, CML is pre-eminently suited for developing an innovative patient-centered care model.

## Methods

### Overview of the Study Design

**Figure 1:**
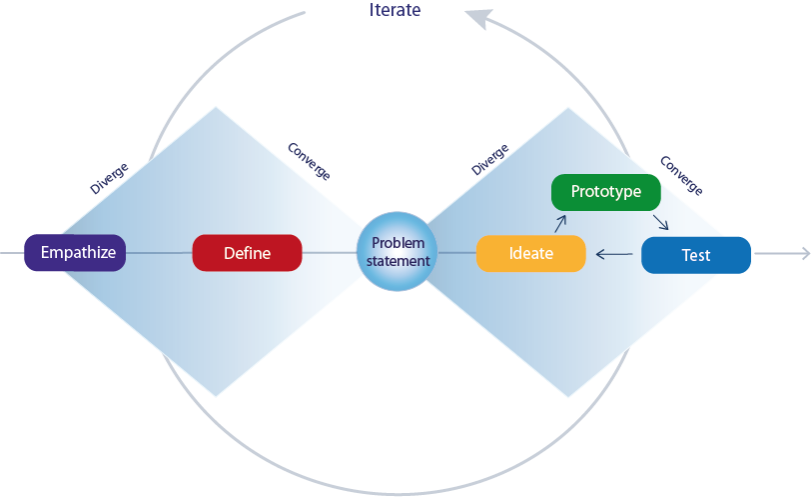
The double diamond model depicting the essentials of the design thinking process.

The design thinking methodology [28] was used for the development of the innovative patient-centered care model. It comprises the following 5 phases: empathize, define, ideate, prototype, and test. We started with the key element of every phase, ie, the end user, and in our case, the patient. Pilot evaluations of the developed innovation were performed by focus group interviews with patients. Design thinking is a dynamic process where phases can run parallel or in iterative circles based on new insights or feedback. Going back is possible at any moment during the process. Generally, it can be visualized in the shape of 2 diamonds: first diverge in empathizing, then converge and define a problem, then again diverge and converge, generating ideas for a solution and prototyping and testing the right solution, respectively. Figure 1 represents this process and is adapted from an illustration developed by Jasper Liu [29].

### Setting

The project has been established by a collaboration among hematologists at 3 hospitals in the Netherlands, the Dutch hematological patient advocacy group, *Hematon*, and the Reshape and Innovation Center of Radboudumc. A multidisciplinary project team was formed to coordinate the process, comprising patients, health care professionals, designers, developers, and a communication specialist, thereby representing both the participating and the developing parties. The study was approved by the institutional review board of the Radboudumc.

#### Phase 1: Empathize

The understanding of the end users and their needs, desires, and challenges in everyday life is crucial and should be explored. To do so, we conducted in-depth interviews with patients and hematologists until data saturation was reached. The interviews were conducted by the user-centered designers of the project team. The patients were asked to participate during a national meeting of the patient association *Hematon* or were approached by their hematologists. The hematologists were approached via professional associations. Furthermore, field observations of different parts of the patient’s journey in the health care system were made. These observations took place in pharmacies, in waiting rooms, during blood sample collection, and at the outpatient clinics during consultations with the hematologists. A checklist was used to evaluate a certain set of items (eg, providing information on prescription medication, waiting time). Communication science students conducted the observations and were supervised by the project team. All information gathered in this phase was clustered into themes to identify connections. Furthermore, a stakeholder analysis was made, with the aid of stakeholder mapping, to identify all stakeholders.

#### Phase 2: Define

During this analyzing phase, all information gathered in the *empathizing phase* was translated into a problem statement: an expression of the problem we were aiming to address. The statement had to be human-centered, broad enough for creative freedom and narrow enough to make it manageable [30]. Therefore, tools such as affinity clustering, the 5 whys, frameworks, and creating a point of view were used [31-33]. The statement was defined by the project team in an iterative process until consensus was achieved.

#### Phase 3: Ideate

Here, the goal was to generate the broadest range of ideas for design as possible, preferably from different stakeholders’ perspectives. We started ideating with a selection of the project team comprising at least a designer, patient, developer, and health care professional. Subsequently, the ideas were shared and modified by the entire project team. By asking “how might we” questions, the information gathered in the previous phases was converted into requirements of the innovation. It was key to strive for as many ideas as possible to solve parts of the problems and not just for one idea to solve the whole problem. In this manner innovation concepts could be formed and tested on a small scale.

#### Phase 4: Prototype

We started with rapid prototyping by developing many low-resolution (quick to make and without much expenses) prototypes, eg, screenshots of possible apps and websites. These were tested early in the process and immediately refined after feedback from the end users and stakeholders. Feedback was collected during the test sessions with small groups of end users. In addition, meetings were held with the project team, complemented by the representatives of different stakeholders. If additional information was found to be missing or new ideas came up, the previous phases were repeated.

#### Phase 5: Test

This phase ran partially parallel with the prototype phase where every feature from the mock-up to concept product was tested iteratively with the end users. Eventually, a pilot test was run on a small scale at the Radboudumc (the center where the project team was situated). A stepwise workflow was developed, containing the tasks and requirements that had to be fulfilled at each step and the person who had to fulfill these in the first 12 months. To create awareness of the innovation, a communication plan was developed for both patients and health care professionals. In the prototype phase, the prototypes were tested in small groups of mostly patients (4-6 patients). During the pilot test, focus groups were conducted with the help of a structured interview guide (Multimedia Appendices 1 and 2). They were conducted with groups of patients and hematologists. Participants were approached via the website of the innovation, *Hematon,* and professional associations. The first focus group comprised 4 patients and 1 patient’s caregiver (3 males and 2 females, aged 54-72 years). In the subsequent focus group, 7 patients participated. They were interviewed about the introduction to and awareness of the innovation, practical issues in utilizing it, layout, content, and the added value in one’s own life. Another focus group was conducted with 13 hematologists, following a CML-focused national working group. The topics were similar to those in the patients’ focus groups (see Multimedia Appendix 2 for the interview guide). Quantitative data on the usage of the innovation’s website and different mobile features were established at 12 and 16 months using Google Analytics and data from the third parties involved, respectively.

## Results

### Phase 1: Empathize

Overall, 8 patients (data saturation was reached) were interviewed to understand their experiences and explore their needs and desires. The mean age of these patients was 53 years (range 28-75), and 75% (6/8) were male. Each interview lasted between 60 and 90 min. The patients’ strongest wish was to be cured of the disease; however, 3 other wishes were mentioned almost unanimously. First, insights into their disease, with more knowledge and comprehension, were desired (for instance, “Am I doing well or not?” and “Where am I in my disease course?”). Second, they wished for better support in understanding and coping with the symptoms they experienced (eg, “I’m having a hard time summarizing all the symptoms of the past 3 months before consultation” and “How can I explain [to] my family and friends what the impact is on my daily life?”). Finally, they wished for improvements in the organization of care delivery. The journey to the hospital and different appointments (ie, hematologist, nurse specialist, blood sample collection, pharmacy) at different times and multiple locations were considered to be time-consuming, apart from the time spent in a waiting room.

A total of 14 hematologists (9 working at a university hospital and 5 at a nonuniversity hospital) were interviewed, and they mentioned the following desires: to empower the patient, better insight into the patient-reported experiences and outcomes, improvement of guideline adherence, and providing care only when medically needed or when desired by the patient.

In addition, 34 observations (data saturation was reached) in the outpatient clinic, during blood testing, or in the pharmacy were made to fully understand the patients’ journey. The observations were not connected to the patients interviewed and took place during different parts of the day. Lengthy waiting times were the biggest issue in almost all observations as well as receiving too little information regarding the prescription medication at the pharmacy. Altogether, the role of the patients was too small in their own care process, and adequate tools to take the lead were lacking.

The following stakeholders were identified: the patients as end users; health care professionals such as hematologists, general practitioners, and, to a lesser extent, pharmacists; molecular biologists; and health insurers.

### Phase 2: Define

The objective of the innovation was defined as “to empower patients and facilitate them to take the lead in their own care process.” As CML cannot be cured, we could not meet the biggest desire of curing the disease. However, it has recently become clear that it is feasible for patients to achieve a durable remission of the disease after discontinuation of their treatment [34,35]. This is referred to as *treatment-free remission* (TFR) and is considered to represent an *operational cure*. TFR is having a stable, deep molecular response without any ongoing TKI treatment. Patients in chronic phase CML who have maintained a stable and deep molecular response (for at least two years) are considered for TKI discontinuation. In an international consensus statement of patient representatives and doctors, TFR was adopted as a desirable goal of CML therapy in the context of adequate medical and psychological support [36]. It was established that optimizing guideline adherence and therapy compliance are prerequisites for achieving this goal.

### Phase 3: Ideate

On the basis of the Define phase, the innovation should provide reliable information and instruments for the patients to gain insights into their own disease course and educate them in the subsequent steps. In addition, the innovation had to support patients when experiencing symptoms, whether disease-related or TKI-related. To reduce logistic struggles such as waiting times and to achieve availability for every patient with CML, the multidisciplinary project team decided that the innovation had to transfer part of the current care from the hospital to the personal environment of the patient.

Owing to the variety in problems, a variety of solutions were proposed, for instance, electronic health (eHealth) technologies to enhance knowledge and comprehension of the disease, provide a way to report and log symptoms, facilitate contact among patients for both social support and sharing insights, improve the organization of care delivery, and eliminate unnecessary steps, and, in addition, a nurse specialist in both CML and electronic nursing, who can empower and educate patients with the aid of eHealth technologies. The multidisciplinary project team came up with a web-based innovation covering these different solutions to achieve the main goal of empowering the patient and making CML care more patient-centered by improving the quality of life, medication adherence, guideline adherence, treatment outcomes, and reducing organizational burdens of the care process.

### Phase 4: Prototype

**Figure 2:**
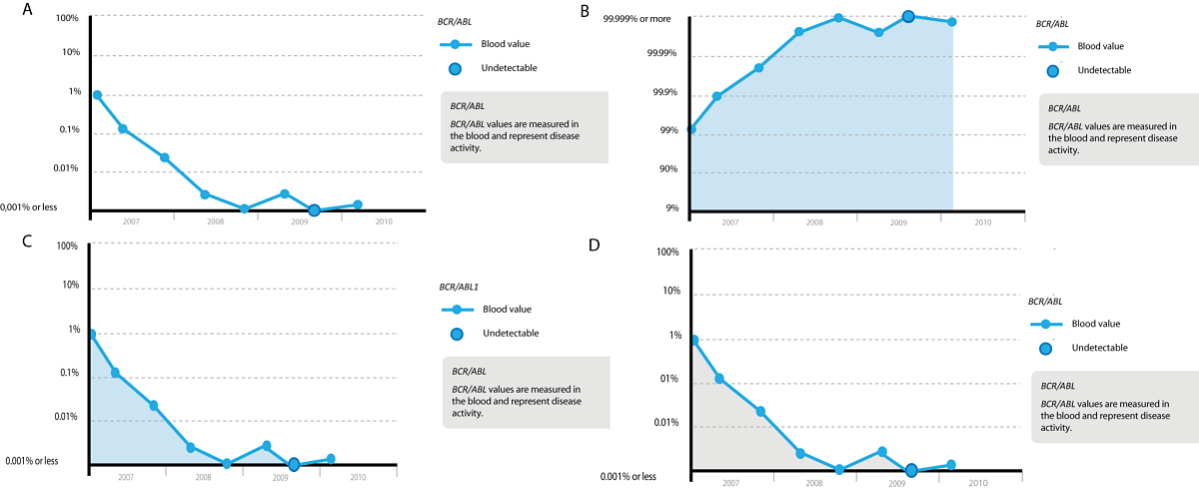
Four examples of graphs depicting BCR-ABL1 values. Graphs B, C, and D have a colored area under the curve, in contrast to graph A. Furthermore, the percentages on the y-axis differ: graphs A, C, and D have percentages from 100 to ≤0.001, representing the number of BCR-ABL1 transcripts. Graph B has a positive approach with percentages from 9 to ≥99.999, representing the amount of healthy cells.

**Figure 3:**
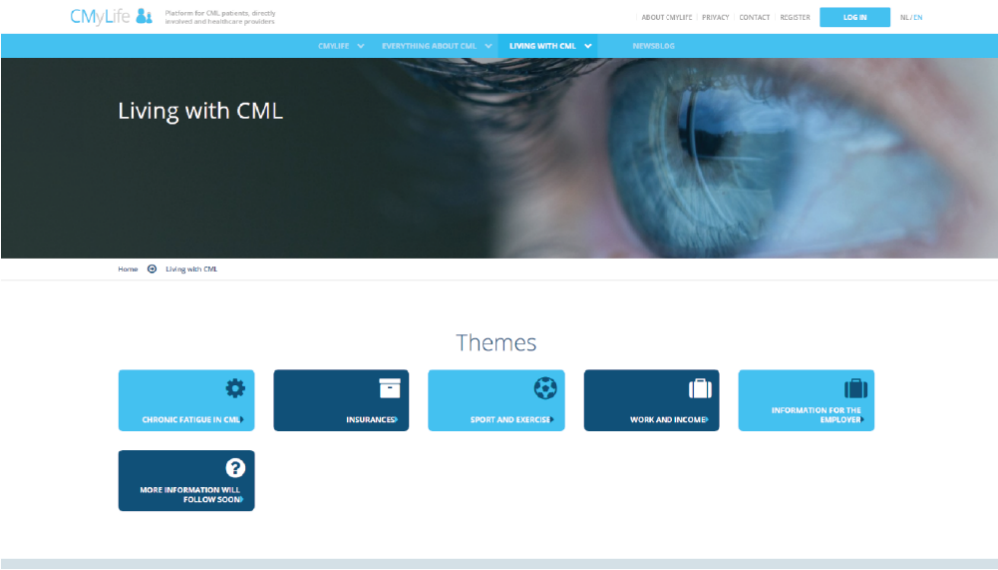
Screenshot of the CMyLife website.

Low-profile prototypes varying in content and layout were made of various parts of the coordinating solution (eg, different graphs depicting *BCR-ABL1* values to select the most insightful one; Figure 2). Eventually, the iterative testing of the prototypes with the patients’ focus groups resulted in the concept of a web-based platform called CMyLife, literally meaning *see my life* and referring to CML. The platform was extended by adding different features, with the patients deciding which features to use in their own care process. It comprised a public website containing reliable information on the disease; treatment (eg, AE profiles per TKI and TFR); other medical issues; the impact on social aspects, eg, sports and financial issues (Figure 3); and a patient-tailored part with additional features in a secure environment. These features have been described as follows.

#### Forum

A forum was developed in a secured part of the platform, where patients could meet other patients and ask questions. It was monitored by 2 patient-experts and the community manager of the project team who can refer the questions to a hematologist, in case of medical questions.

#### Patients Know Best

Another feature is the Patients Know Best portal [37], where patients are in control of their personal medical record. They can grant access to the medical records to their health care professionals (including professionals other than hematologists) or choose to keep them private. For patients in the pilot hospital, a connection was made between the hospital’s eHealth record and the Patients Know Best portal. In this way, laboratory results are uploaded to the Patients Know Best automatically.

#### MedApp

We reached out to the developers of the mobile phone app, MedApp, aimed at helping patients managing their medications [38]. This resulted in a *CMyLife module* that is linked to a patient’s Patients Know Best portal in the app. In this module, patients can rate 10 frequent TKI-related symptoms daily on a 4-point Likert scale, resulting in a weekly or monthly overview. This can be used as a visual representation of the impact of CML on their daily life and can be shared with family and friends. If the patients share their Patients Know Best record with the hematologist, this can result in a more tailored consultation. Preparations are made to develop a feedback loop signaling the nurse specialist to contact the patient in case of grade 3 or 4 AEs.

#### Disease Activity

**Figure 4:**
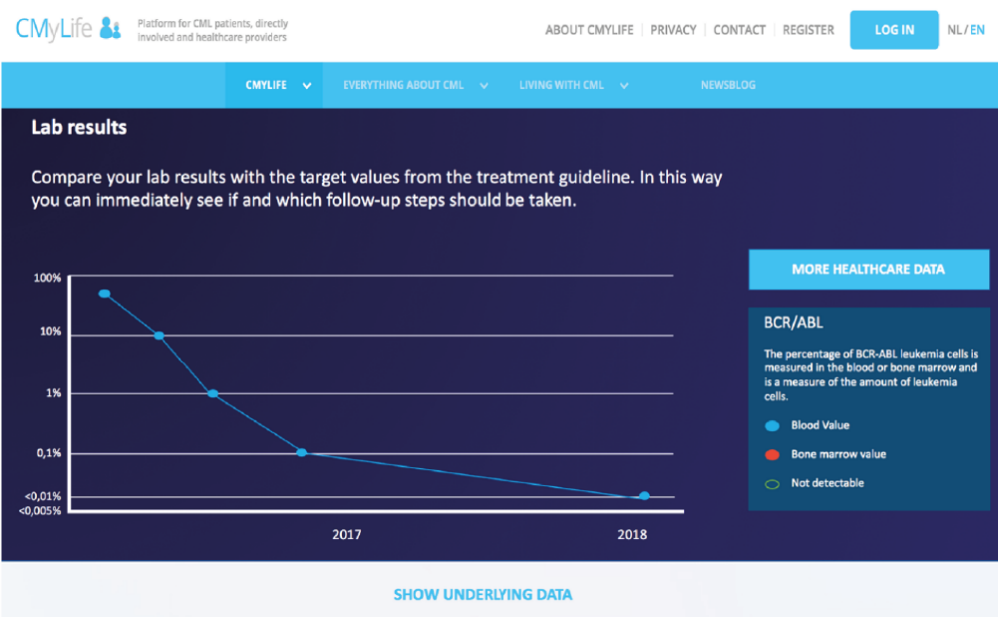
Screenshot of the graph depicting BCR-ABL1 transcript values in a patient’s own Patients Know Best record in the CMyLife environment. When a dot is selected, it shows the results and its date.

To provide insight into an individual’s disease course, graphs depicting the molecular marker measured to monitor disease activity (*BCR-ABL1* transcript values) are uploaded in the Patients Know Best record, in collaboration with the local molecular laboratory (Figure 4).

#### Guideline Application

The above-mentioned graph (Figure 4) visualizing the molecular marker levels is linked to the guideline app. This app contains an easy to understand stepwise explanation of the Dutch CML guideline for patients with CML in chronic phase using a first-line TKI [39]. On the basis of their last molecular response and moment of testing, patients receive advise, for example:

The response is optimal, you should continue with your medication.

The response is not optimal. This can be due to medication interactions, skipping medication or progression of disease. Ask your hematologist about it on your next appointment.

In addition, reminders are sent when patients should be tested again. Expanding the functionalities to second-line treatment and CML in other phases is planned for the near future.

#### Reducing Hospital Visits

Furthermore, organizational issues were addressed by the following features, all aimed at minimizing hospital visits.

##### Blood Samples

In collaboration with the Thrombosis Service, a service monitoring anticoagulant therapy in the Netherlands, which draws blood samples at home or nearby for patients with thrombosis using oral anticoagulants, patients with CML can get their blood samples drawn at home.

##### Pharmacy

TKIs are delivered to the patient’s home by the pharmacy. In the MedApp’s CMyLife module, patients can request the pharmacy directly for a new delivery when they are almost out of TKIs. Hence, a 12-month valid prescription is supplied by the hematologist.

##### Screen-to-Screen Consulting

Finally, patients are offered a screen-to-screen consultation with their hematologists. Consultation rooms were provided with the right equipment, and health care professionals were trained. Face-to-face consultations with the hematologist were reduced to once a year unless additional face-to-face physical consultations at the hospital were considered desirable by the patient. Between these visits, patients were supported through screen-to-screen consultations and the other features described above.

#### Security

All data were secured, and data protection conformed to the national and European privacy regulations (General Data Protection Regulation). The features containing data from the hospital’s electronic health record (ie, laboratory results in their personal health record, website, and app) were secured with a password-protected log-in and subsequent token via an SMS text message for authentication. This is in accordance with the Dutch security guideline NEN7510. The forum is password protected.

Before the connection can be made between the hospital’s systems and CMyLife, a patient has to register for a personal health record. Subsequently, their identity is checked based on name, date of birth, postal code, and citizen service number. The personal medical record is International Organization for Standardization/International Electrotechnical Commission 27001:2013 certified.

### Phase 5: Test

#### Focus Groups

Patients had different experiences of how and when CMyLife was introduced to them. Some preferred an introduction by the patient association *Hematon*, others by the hematologist. With regard to the practical issues in its utilization, it turned out that patients had little experience with the platform at this time (“I did not even know it existed”). A few content-specific suggestions were made, such as a small piece of information helping patients explain CML and its impact to others. Screen-to-screen consultations and having blood samples drawn at home received a positive response:

I really like the video-consulting, this way I am certain I don’t have to waste a half day every 3 months or so.

My consultations with my doctor never last longer than 3 minutes. “Everything looks fine, great, bye and see you next time.” (...) What am I doing here. There are people who need the hematologist more than I do, especially when I’m doing well. I would prefer video-consulting, it saves time for the both of us.

I receive a prescription from my doctor, go to the hospital pharmacy and a few days later I can pick up my TKIs, if I am lucky...And you always have to make a call on forehand to check if your order is ready. It is old-fashioned.

Insights into individual laboratory results was desirable when combined with an easy-to-understand version of the guidelines (“We do not want our care to be dependent of which doctor is treating you, it should be the same everywhere”). The responses to registering symptoms and medication intake were variable as everyone could not imagine this to be beneficial to their management:

It involves extra steps and it can result in more work, though.

I understand the advantage, a doctor can prepare the consultation.

Hematologists were a little more reluctant regarding CMyLife. They could imagine the added value in improving patient empowerment and transferring care to outside the hospital. However, there were concerns regarding time investment, as time is a scarce resource in daily practice:

I think it is very important to provide care at home and for a patient to take the lead. The problem is that as a doctor, I don’t want to have extra work, so it has to be very well facilitated.

I think it can help improving adherence. Of course, patients only experience CML-related symptoms if it went wrong, if patients monitor their own results and see the numbers and decrease in log value, it could be stimulating to be really adherent to their medication.

All our patients search the internet for information. What you want as doctors is provision of trustworthy information, I think that is an important goal of such a CMyLife intervention.

I see potential; however, I also foresee many technical issues in connecting the CMyLife system to our hospital system.

Through a system like this, you can diminish a lot of routine follow-up visits every 3 months, in order to create more time for other things.

Another topic of concern was the growing development of (commercial) mobile interventions, all specified to just a specific disease and often focused on just one aspect of that disease:

An important barrier is the coordination with similar initiatives for other patients than CML patients. One universal solution is desirable. The strength of CMyLife is that it can be regarded as a blueprint that can be used for other diseases.

Patients have comorbidities, some patients see a hematologists, cardiologist and other doctors, it would be preferable if CMyLife was not a stand-alone application.

#### User Statistics

With regard to the user statistics of the CMyLife website, in the first year (December 2016-December 2017), CMyLife had 6334 unique visitors, with approximately 600 visitors per month for the last 6 months. Unique page views in the last 12 months were 36,300. Per session, 5.17 pages were visited on average, with the page containing the latest blogs and information on AEs having the most visits after the homepage and log-in page. A total of 338 users were registered, who were treated in 62 different hospitals. Per month, the forum was visited on an average of 150 times by 40 users. One-fifth of the visitors were new; 80% comprised returning visitors. A total of 677 users registered for the CMyLife module in MedApp, with 156 active users (ie, monthly use) in the last month.

#### Running and Expanding the Pilot

The publicly available website and online user forum were launched nationwide during *Hematon*’s information day on CML, on April 23, 2016. Gradually, we extended the other features for patients of the Radboudumc to test and refine CMyLife and prepare for further implementation. The workflow of the electronic consultations has been described hereafter. As the pilot was confined to patients of the Radboudumc, the additional features (ie, except the website and user forum) were separated from the public version of the tool.

Patients received a letter from their hematologist accompanied by a CMyLife information brochure. Extra time was scheduled for their next follow-up visit. This visit comprised 4 parts. First, the hematologist completed the routine follow-up and then explained the changing model of care. If desired, a 12-month valid prescription was written for TKIs to be delivered to the patient’s home. Second, an introduction with the nurse specialist took place, followed by an explanation of the functions and first half of the features of CMyLife (information source and Patients Know Best). Third, the patient visited a member of the project team who showed an explanatory video and installed and activated CMyLife and Patients Know Best (after an identification check). If necessary, instructions were provided by email and telephone. In total, 80 min were reserved for these 3 parts. Finally, the patient visited the pharmacy where the type of medication and the number of pills in possession of the patient were registered in CMyLife. After 3 months, the patient had a routine follow-up scheduled with the hematologist, who could discuss the symptoms reported in CMyLife and TKI adherence. Thereafter, the patient visited the nurse specialist who evaluated the experiences of using CMyLife and explained the second half of the features (ie, video consulting, having blood drawn at home, and scoring symptoms). Both visits lasted for 20 min each. After 6 months, the hematologist proposed the possibility of screen consulting for the next visit, followed by an explanatory visit, including instructions for video consulting with the nurse specialist.

## Discussion

### Principal Findings

Finally, with the aid of the design thinking methodology, the CMyLife innovation was developed, which is a web-based platform with multiple features such as a website with reliable information and a forum, a guideline app, personal medical records with logs of symptoms and laboratory results (including molecular markers connected to the guideline app), screen-to-screen consulting, and delivery of medication and collection of blood samples at home. Since the beginning of the pilot, 338 users were registered, and the website had 6334 unique visitors. However, it should be noted that CML is a rare disease, with approximately 160 new patients per year in the Netherlands.

Gaining insights into the course of one’s disease was most desired by patients. Hence, an easy-to-understand version of the Dutch CML guideline connected to personal laboratory results was developed, which provides patients with the tools to take the lead in their own care process, enhancing patient empowerment and guideline adherence. In daily practice, adherence to guidelines by physicians is often suboptimal [18-20,40,41], which is associated with lower medication adherence and inferior treatment outcomes, including an increased risk of progression to an advanced phase of the disease [19-21,24]. A second feature is the provision of trustworthy information on a variety of subjects related to CML care, based on patients’ feedback. Furthermore, by transferring the majority of care outside of the hospital, CML care is centralized digitally. This could be beneficial as adherence to guidelines was found to be the lowest in small centers treating just one or a few newly diagnosed patients with CML [42]. Even for patients who have achieved TFR and discontinued TKI treatment, CMyLife could be of added value. As approximately half of the patients remain in TFR after TKI discontinuation [43-45], optimal monitoring is important.

Another key feature of CMyLife is the personal health record that can be created and filled with patient-reported outcomes (PROs) such as TKI intake, health status, and AEs. In the literature, AEs are frequently reported and are even described as the main reason for skipping TKI doses intentionally [46-49]. These low adherence rates are a serious challenge in CML care [48,50,51], leading to decreased treatment responses and subsequently increased rates of resistance and progression [22,23,47,52]. Obviously, recognition of AEs by the physician is essential to relieve the symptoms or consider switching TKI to enhance adherence. However, physicians tend to rate symptom severity lower than patients and overestimate the adherence of their patients [27,53]. Patients reporting their own outcomes have improved control of symptoms, medication adherence, and patient satisfaction. Furthermore, PROs provide distinct prognostic information, exceeding clinical measurements [54-56].

Naturally, the utilization of human-centered design approaches and the development of mobile health technologies in health care are not new. There are numerous examples of mobile health technologies for patients and their caregivers for various diseases. Even interactive patient portals in breast and lung cancer care, developed with active patient participation, are described in the literature [57,58]. Laboratory result, PRO, and frequently asked question sections are common features of these portals. Other web-based interventions include a previsit communication tool for malignant lymphoma, an online community for adolescents and young adults with cancer, online communities for patients with breast cancer, and a web-based app supporting cancer survivors in self-management by monitoring PROs and acquiring feedback with a summary of personalized supportive care options [59-62]. As such, the individual web-based features of CMyLife are not unique; however, the combined features and the transfer of care from the hospital to the patient’s own environment are unique. CMyLife changes the current delivery of care and centralizes it digitally.

Laboratory results, PROs, electronic consulting, and information provision can be considered as generic components of innovative apps for all types of cancer and even for nonmalignant chronic diseases. It is key to the development of such innovations to start at the beginning, ie, with the patients, and tailor the innovation as a solution to the patients’ needs. CMyLife in its entirety incorporates a holistic approach, which could serve as an example for innovations for other malignancies.

CML, however, is distinguishable from other malignancies based on the nearly normal survival rates and, above all else, the availability of a molecular marker representing disease activity. Fortunately, survival is nowadays seldom the key issue for patients and the emphasis shifts to the management of AEs, decreasing the disease burden in daily life.

### Limitations

Design thinking was an effective method to develop an innovation in a bottom-up approach. However, it is possible that a different product would have been developed if another user-centered method had been used. In addition, our study is limited by the possible bias in patients voluntarily participating in the development of the innovation. Patients willing to participate in the project could be a more active group, and their needs may not represent the needs of the overall population of patients with CML.

### Future Research

The ability of CMyLife to achieve its goal as well as the added value of CMyLife and its features will be the focus of future studies. Pre- and posttest design studies were running at the time of writing this paper. CMyLife will be evaluated on objective criteria (ie, quality of life, cost-effectiveness, adherence rates, etc). In addition, the further development and fine-tuning of CMyLife remains to be a continuous process. During this process, qualitative studies with more focus groups and interviews will be performed.

Enhancing adherence could save health care costs. A Dutch report assessed the potential value of improving both patients’ adherence to TKIs and physicians’ adherence to guidelines [63]. For each yearly cohort of newly diagnosed patients with CML (160 patients) over a period of 25 years, a cost reduction of approximately US $3.1 million (€2.8 million) and US $1.3 million (€1.2 million) would be possible by improving the patients’ adherence and physicians’ adherence, respectively, to 100%. For the improvement of every 1% of adherence, cost reduction was established at US $118,460 (€106,560) and US $48,380 (€43.520) per yearly cohort over 25 years, respectively.

### Conclusions

The main takeaway of this study is that we have developed a holistic intervention with the aid of active patient participation. We aimed to change the current model of care by using and continuously improving CMyLife. Patients are provided with an easy to understand guideline for the management of CML. In addition, they receive their own laboratory results, including the paramount *BCR-ABL1* values, depicted in a simple yet understandable graph. With the aid of CMyLife, we give patients the tools to monitor their results, interpret these results, and act on them. They are provided with the know-how to consider their results in relation to their personal care process. Patients themselves monitor their treatment and know when it is time to check their values again. After all, physicians proved not to be the most adherent monitors [41]. Of course, the hematologist will not become completely redundant, monitoring is still required, although from a distance. An approximate comparison can be made with a chronic disease such as diabetes mellitus, where patients monitor their own glucose levels and therapy and have, eg, yearly contact with their health care professional. CML would be the first malignancy where patients are able to monitor and manage their disease by themselves.

Historically, CML has been a prototype malignant disease for innovations in medicine: it was the first human cancer in which the causative genetic abnormality was identified (the Philadelphia chromosome) and was the first disease for which a successful targeted anticancer therapy was developed (the TKI imatinib). Perhaps, CML can lead innovation in oncology once again and be the model of change in current health care by redesigning the model of care to be truly centered around the patient, with the patient in full control and ensuring the highest quality and best outcomes.

## Appendix

Multimedia Appendix 1 Interview guide focus groups patients.

Multimedia Appendix 2 Interview guide focus groups hematologists.

## Abbreviations

AE: adverse event
CML: chronic myeloid leukemia
eHealth: electronic health
PRO: patient-reported outcome
TFR: treatment-free remission
TKI: tyrosine kinase inhibitor
