# Supplementary material for: The Development of a Web-Based, Patient-Centered Intervention for Patients With Chronic Myeloid Leukemia (CMyLife): Design Thinking Development Approach
Source: J Med Internet Res. 2020 May 15;22(5):e15895. doi: 10.2196/15895 (PMC7260663; doi:10.2196/15895)
Supplement: Multimedia Appendix 1 [file jmir_v22i5e15895_app1.pdf]

Interview guide focus group patients.

Short introduction: in order to improve the care for CML patients the platform CMyLife has been developed. It provides answers, support and self-management in order to help you take the lead in your own care process. You can find trustworthy information on the platform, an online community to get in touch with other CML patients, a personal health record with your own lab results and the possibility to ask questions to hematologists.

We would like to start with an introduction round. During your introduction, could you tell us whether you have used CMyLife and if so, whether you do this on a regular basis.

The conversation is divided in 4 parts:

1. Short introduction.
2. Introduction of CMyLife (when and who).
3. Practical use of CMyLife.
4. The added value for you as a patient.

1. Short introduction

Before we start discussing the different features of CMyLife, we would like to hear your opinion of CMyLife as a whole: the sum total of features.

- What is your overall impression/opinion of CMyLife (for instance: positive/negative, useful/useless, fast/time consuming, etcetera)?

2. Introduction of CMyLife
   - At what moment in your care process has CMyLife been introduced to you?
   - What do you think of this moment? What do you think would be the best moment to introduce CMyLife?
   - Who introduced CMyLife to you?
   - Do you think this was the right person to introduce CMyLife? Who would have been a more suitable person? And why?
   - Did you receive any help or explanation regarding the utilization of CMyLife. If so, by whom?
   - Was the amount of explanation satisfactory?
   - What do you think is important when explaining CMyLife? Would you have preferred extra information?

3. Practical use of CMyLife

Usage and login

- Who of you has logged in? Who did not log in?
- How many times have you logged in?

- Who has logged in just once, and did not/barely use CMyLife ever since?
- Who logs in on a regular basis and uses CMyLife?

For the people not/barely using CMyLife

- What is the reason you are not using CMyLife?
- What are the experienced barriers in using CMyLife? (barriers can appear at different levels: CMyLife itself, healthcare professional (HCP), patient, patient's or HCP's environment, hospital organization or financial/legal aspects).
  For instance:
  - Practical problems (e.g. difficulties logging in) (patient)
  - Not knowing how to you use/apply eHealth (patient)
  - Having doubts on the concept of eHealth (patiënt)
  - Having doubts on the content of CMyLife (organization)
  - Lack of time (patiënt)
  - Fear of poor privacy regulations (legal)
  - Missing connections with existing Radboud/other systems (organization)
- What changes have to be made in order for you to use CMyLife?
  - Improvement of the login and usability of the program (organization)
  - Improvement of eHealth knowledge (patient)
  - Increase in eHealth skills (patient)
  - Improvement of the CMyLife content (which topics) (organization)
  - Improvement of privacy and security regulations (legal)
  - Improved communication with existing (Radboud)systems (organization)

For the people who use CMyLife

- What do you like about CMyLife? What do you not like about CMyLife? (*refer to levels mentioned above*).
- Which parts (features) of CMyLife do you use regularly, which ones barely and why (for each feature)?
  - Usability (speed etcetera).
  - Adapt/apply eHealth to own needs and desires.
- What would help you to increase the usage of CMyLife (*each feature*)?

Content

- What is your opinion regarding the content of CMyLife?
- Which topics are useful and which ones are redundant?
- What would you like to have added to the content?

Lay-out

- What do you think of the way the information is displayed? (*each feature*)
- What's your opinion on the ease of retrieving information?
- What do you think of the readability of the information?
- How do you like the interface's lay-out?

Functionality

- What do you think of the speed of the system?
- What is your opinion on the usability of the system?
- What do you think of the possibility to personalize settings in MedApp? And to receive updates on new messages posted on the forum?

4. Added value for you as a patient

One of the goals of CMyLife is to provide more accessible, reliable and understandable information, which fits your personal situation.

- Do you think we have achieved this goal with CMyLife? If not, what could help us to achieve this goal?
- Do you think the information provided by CMyLife is of added value to the information you have already received in the hospital?
- Which information is important to add to CMyLife?

Another goal of CMyLife is to personalize CML care.

- How can the care you receive be more tailored to your personal situation and wishes?

A third goal of CMyLife is to support you in self-management of your disease and treatment, i.e. to put you in the lead of your own care process. The informative website and personal health record could be helpful.

- How can CMyLife help you to manage your own disease?

Could CMyLife contribute to improving your involvement in your own care process? With the aid of, for instance, the information on the website, registering your own outcomes in the app which you can share with your HCP, and insights in your own lab results.

5. Reflection on concrete subsequent steps

We will present four examples of subsequent steps, we would like to hear your opinion regarding these steps.

1. <u>MedApp</u>: with this application you can register your medication intake and adverse events on a scale from 1-4. This information could help your HCP supporting you. By using the MedApp, you could set medication-alerts, log your symptoms and record consultations with your HCP. The information collected can be saved in the personal health record of CMyLife as well.
2. <u>Lab results</u>: in the personal health record in CMyLife you are provided insights in your lab results, depicted in a simple, understandable graph. You would be able to compare the results and interpret them with the aid of the provided understandable guideline on CML treatment.
3. <u>Video consulting</u>: it is possible to replace the periodic consultation with your HCP with a consultation via a secured video connection.

You won't have to come to the hospital, instead you can speak to your HCP through your smartphone/tablet/personal computer. De HCP can share his/her screen, so you can watch with them.

4. Collection of blood in your own environment: your HCP could arrange the collection of blood in your own environment, or even at home. This way, you don't have to go to the hospital. Instead you could go to a local diagnostic center/stay at home.

(Explore reactions and additional ideas regarding the 4 steps).
