# Supplementary material for: The Development of a Web-Based, Patient-Centered Intervention for Patients With Chronic Myeloid Leukemia (CMyLife): Design Thinking Development Approach
Source: J Med Internet Res. 2020 May 15;22(5):e15895. doi: 10.2196/15895 (PMC7260663; doi:10.2196/15895)
Supplement: Multimedia Appendix 2 [file jmir_v22i5e15895_app2.pdf]

Interview guide focus group hematologists.

This meeting explores your experiences with CMyLife and is divided in 4 parts:

1. Short introduction.
2. Knowledge of CMyLife.
3. Practical use of CMyLife.
4. The added value for the patient.

1. Short introduction

Before we start discussing the different features of CMyLife, we would like to hear your opinion of CMyLife as a whole: the sum total of features.

- What is your overall impression/opinion of CMyLife (for instance: positive/negative, useful/useless, fast/time consuming, etcetera)?

2. Knowledge of CMyLife
   - How did you find out about CMyLife?
   - Did you receive any explanation regarding the system?
   - Do you know how CMyLife works?
   - Do you know how CMyLife looks like?

3. Practical use of CMyLife
   - Have patients been receptive to use CMyLife in the daily practice? If so/not, why?
   - What do you think the barriers are in using CMyLife and in the efficacy of CMyLife in daily practice?
     - Not knowing how to you use/apply eHealth
     - Having doubts on the concept of eHealth/the content of CMyLife
     - Lack of time
     - Lack of convenience
     - Fear of poor privacy regulations
     - Missing connections with existing Radboud/other systems
   - What do you think are positive factors regarding CMyLife?
     - Usability (speed etcetera)
     - Adapt/apply eHealth to own needs and desires.

Content

- What is your opinion regarding the content of CMyLife?
- Do you think information is missing?
- Have all the different parts of the care process been discussed sufficiently?

Lay-out

- What do you think of the way the information is displayed? (*each feature*)
- What's your opinion on the ease of retrieving information?
- What do you think of the readability of the information?
- How do you like the interface's lay-out?

Functionality

- What do you think of the speed of the system?
- What is your opinion on the usability of the system?
- What do you think of the possibility to personalize settings?

4. Added value for you as a patient

One of the goals of CMyLife is to provide more accessible, reliable and understandable information, which fits the personal situation of your patient.

- Do you think we have achieved this goal with CMyLife? If not, what could help us to achieve this goal?
- Do you think the information provided by CMyLife is of added value to the information the patient already receives in the hospital?

Another goal of CMyLife is to personalize CML care.

- In your opinion, to what degree has CMyLife supported patients in self-management and having them take the lead in their own care process?
- In your opinion, to what extent are patients more involved in their own care process because of CMyLife?

Other remarks.

- Do you have any other remarks/suggestions concerning CMyLife?
- Thank you for your participation.
